# The dual role of CCND1 in heterotopic ossification: A Non-canonical Pathway for Celecoxib treatment

**DOI:** 10.1016/j.heliyon.2024.e34936

**Published:** 2024-07-20

**Authors:** Wei Liu, Junchao Huang, Jianhai Hu, Ziheng Bu, Zheng Zhou, Jianing Yu, Huajun Wang, Xinbo Wu, Peng Wu

**Affiliations:** a Department of Orthopedics, Shanghai Tenth People's Hospital, School of Medicine, Tongji University, Shanghai, 200072, China; b Department of Sports Medicine, The First Affiliated Hospital, Guangdong Provincial Key Laboratory of Speed Capability, The Guangzhou Key Laboratory of Precision Orthopedics and Regenerative Medicine, State Key Laboratory of Frigid Zone Cardiovascular Diseases, Jinan University, Guangzhou, 510630, China

**Keywords:** Celecoxib, Heterotopic ossification, Network pharmacology, Molecular docking, CCND1, Rheumatoid arthritis signaling pathway, NF-Kappa B signaling pathway

## Abstract

**Objective:**

To explore the effective targets of Celecoxib in the treatment of heterotopic ossification using network pharmacology methods.

**Methods:**

Potential molecules related to heterotopic ossification were obtained by retrieving the GEO and CTD databases and intersecting them. Potential binding targets of Celecoxib were acquired from the STITCH database. A protein-protein interaction network was constructed between potential binding targets of Celecoxib and potential related molecules of heterotopic ossification using the STRING database. Molecules in the protein-protein interaction network were further analyzed using GO and KEGG enrichment analysis in R software, followed by enrichment analysis of active molecules in the Celecoxib-heterotopic ossification target dataset. Hub genes were selected based on the “degree” value and enrichment within the protein-protein interaction network. The binding affinity of hub genes to Celecoxib was observed using molecular docking techniques. Finally, in vitro experiments were conducted to validate the effectiveness of hub genes and explore their regulatory role in the progression of heterotopic ossification. Additionally, the therapeutic effect of Celecoxib, which modulates the expression of the hub genes, was investigated in the treatment of heterotopic ossification.

**Results:**

568 potential molecules related to heterotopic ossification and 76 potential binding targets of Celecoxib were identified. After intersection, 13 potential functional molecules in Celecoxib's treatment of heterotopic ossification were obtained. KEGG analysis suggested pathways such as Rheumatoid arthritis, NF-kappa B signaling pathway, Pathways in cancer, Antifolate resistance, MicroRNAs in cancer play a role in the treatment of heterotopic ossification by Celecoxib. Further enrichment analysis of the 13 potential functional molecules identified 5 hub genes: IL6, CCND1, PTGS2, IGFBP3, CDH1. Molecular docking results indicated that Celecoxib displayed excellent binding affinity with CCND1 among the 5 hub genes. Experimental validation found that CCND1 is highly expressed in the progression of heterotopic ossification, promoting heterotopic ossification in the early stages and inhibiting it in the later stages, with Celecoxib's treatment of heterotopic ossification depending on CCND1.

**Conclusion:**

In the process of treating heterotopic ossification with Celecoxib, immune and inflammatory signaling pathways play a significant role. The therapeutic effect of Celecoxib on heterotopic ossification depends on the hub gene CCND1, which plays different roles at different stages of the progression of heterotopic ossification, ultimately inhibiting the occurrence of heterotopic ossification.

## Abbreviations and acronyms

ARS: Alizarin red staining
ALP: Alkaline phosphatase
BP: Biological Process
CC: Cellular Component
CTD: Comparative Toxicogenomics Database
GEO: Gene Expression Omnibus
HO: Heterotopic ossification
IGFBP3: Insulin-like Growth Factor Binding Protein 3
MF: Molecular Function
PPI: Protein-Protein Interaction
PDTC: Pyrrolidine dithiocarbamate
PTGS2: Prostaglandin-Endoperoxide Synthase 2

## 1 Introduction

Heterotopic ossification (HO) represents a pathological condition characterized by the aberrant formation of mature bone in atypical locations, disrupting skeletal muscle homeostasis and regeneration [1]. This phenomenon is predominantly attributed to genetic predispositions or as an acquired consequence of trauma [2]. The clinical manifestations of HO vary depending on its developmental stage. In the initial phases, patients typically present with localized swelling, pain, and tenderness. As the condition progresses, mobility in the affected joints may decrease, potentially leading to complete ankylosis in severe cases. This significantly impairs the quality of life for patients and poses a substantial threat to their daily activities and long-term health [3]. Therefore, an in-depth study of the treatment and prevention strategies for HO can not only significantly improve patient prognosis but also provide new perspectives and strategies for research in the field of musculoskeletal diseases.

Celecoxib, as a highly selective NSAID, has shown significant therapeutic efficacy in the treatment of HO [4]. Its mechanism of action primarily involves targeted inhibition of cyclooxygenase-2, effectively suppressing the production of inflammatory prostaglandins in the early stages of trauma, reducing local inflammation, and preventing the transformation of mesenchymal stem cells into osteoblasts, thereby preventing the onset of HO [5,6]. The therapeutic effectiveness of Celecoxib is comparable to that of the commonly used Indomethacin, but it has the advantage of causing fewer gastrointestinal side effects [7,8]. Therefore, Celecoxib is recommended as the preferred medication for patients without cardiovascular diseases.

While the medical community widely acknowledges that the therapeutic efficacy of Celecoxib is primarily due to its inhibition of cyclooxygenase-2, the therapeutic effects of drugs typically involve multiple targets and mechanisms. Therefore, further exploration of the mechanisms underlying the therapeutic effects of Celecoxib is essential. This not only helps to deepen our understanding of its role in the treatment of HO but also provides more targeted guidance for future clinical therapy.

Network pharmacology, an emerging discipline grounded in systems biology, conducts network analyses encompassing "drugs, diseases, genes, and targets," delving into the intricate relationships among the numerous targets involved in drug therapy for diseases [9]. Leveraging computational analysis technologies, it facilitates the construction of a "component-target-pathway" network, enabling a systematic examination of the interactions between drugs and diseases on a holistic scale. This approach permits the application of a molecular biology perspective in uncovering the potential functionalities of drugs in disease treatment [10]. Its profound significance lies in the elucidation of drug action mechanisms, paving the way for groundbreaking research and development in drug innovation.

**Fig. 1:**
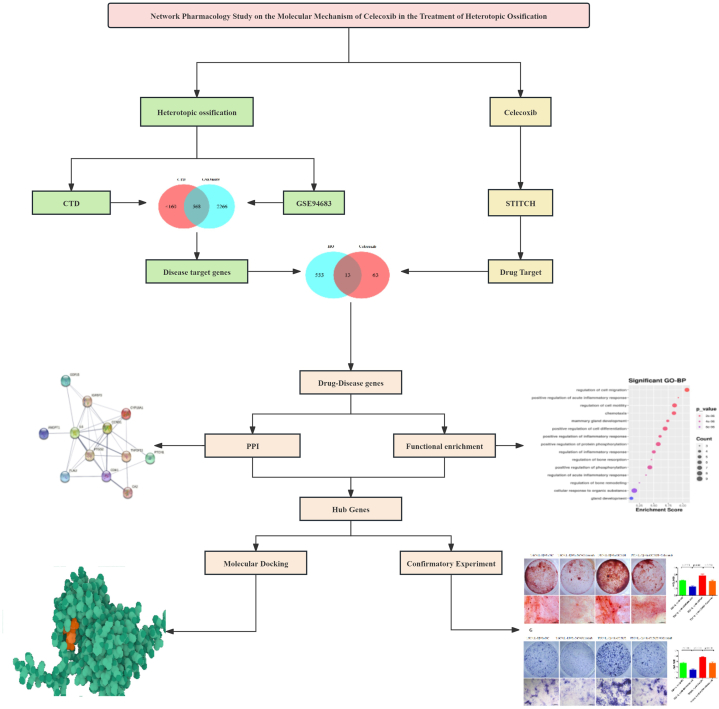
Workflow diagram of method.

Contemporary research into the mechanism of Celecoxib's effectiveness in treating HO often presents a one-dimensional and static viewpoint. However, the action of Celecoxib on HO is likely characterized by its interaction with multiple targets and signaling pathways. To address this complexity, we have employed network pharmacology methods, aiming to unravel as many comprehensive mechanisms as possible by which Celecoxib impacts HO. Additionally, we have conducted a series of experiments for empirical validation, including RT-qPCR, Alizarin red staining, and Alkaline phosphatase staining. (Fig. 1). Through this approach, we anticipate providing more effective treatment options for patients with HO, while also contributing new theoretical and practical foundations to the scientific research in the field. Our study will not only enrich our understanding of the pathophysiology of HO but also provide more precise guidance for clinical treatment, with the potential to reduce side effects of medications and improve therapeutic outcomes. Furthermore, the methodology and findings of this research will serve as a reference for multi-target studies of other drugs, promoting the application of network pharmacology in drug development, and accelerating the discovery of new drugs and the development of new uses for existing medications.

## 2 Methods

### 2.1 Data profile

Initially, we identified target genes associated with Celecoxib by querying the STITCH database. Subsequently, we accessed and downloaded genes related to HO from the Comparative Toxicogenomics Database (CTD), ensuring that the data obtained pertained specifically to the Homo sapiens, available at http://ctdbase.org/. Finally, we obtained gene expression data from the Gene Expression Omnibus (GEO) database, selecting the dataset GSE94683 (Homo Sapiens) for our analysis.

### 2.2 Differential expression analysis

Initially, differentially expressed genes in patients with HO were retrieved from the GEO database, accessible at https://www.ncbi.nlm.nih.gov/. This was followed by the application of the limma package [11] in R language for computation and analysis. The selection criteria for heterotopic ossification-related differentially expressed genes included an absolute value of Log Fold Change greater than 1 and an adjusted P-value less than 0.05.

### 2.3 Construction of the drug–target–disease network diagram

Initially, the task involves identifying target genes associated with the drug of interest. Following this, we pinpoint the intersection genes between the disease-specific target genes and the differentially expressed genes. The final step encompasses identifying the intersection of drug target genes and disease target genes. This information is then utilized to construct a comprehensive drug-target-disease network using Cytoscape software.

### 2.4 Gene functional enrichment analysis

This study performed functional enrichment analysis of candidate genes utilizing the Gene Ontology Database [12] and KEGG pathway database [13]. A statistical algorithm, specifically Fisher's exact test, was employed to ascertain which functional categories are most pertinent to the given gene set. In the analytical results, each category is associated with a level of statistical significance, denoted by a P-value. A lower P-value indicates a stronger association between the functional category and the input genes, suggesting that a majority of the genes in this set are characterized by the functionalities corresponding to the identified category.

### 2.5 Protein-protein interaction (PPI) network construction

In this experiment, the online tool STRING [14] was utilized for analyzing the PPI among the candidate genes, setting a required confidence threshold (combined score) of >0.4 for the interactions. The topology of the PPI network was analyzed using Cytoscape, based on the established PPI pairs. Observations from the constructed biological networks indicate that they predominantly adhere to the characteristics of a scale-free network. Consequently, by employing connectivity degree analysis within network statistics, it was possible to identify key nodes actively involved in protein interactions within the PPI network, specifically the hub genes [15]. Furthermore, this study conducted node analysis and leveraged the scale-free nature of the PPI network to identify central proteins within the network.

### 2.6 Molecular docking validation

In our study, we employed AutoDock Vina software to intricately analyze the binding affinity and interaction patterns between Celecoxib and key target genes. The molecular structure data for Celecoxib was sourced from the PubChem Compound database. Concurrently, the three-dimensional coordinate data for the hub genes were obtained from the PDB database. To facilitate docking analysis, all protein and molecular files were converted to PDBQT format, with all water molecules removed and polar hydrogen atoms added. The grid box was set at a central position, covering the region of each protein and allowing free movement of the molecules within. The grid box dimensions were established at 30 Å × 30 Å × 30 Å, with a grid point distance set at 0.05 nm. Ultimately, molecular docking studies were conducted using version 1.2.2 of AutoDock Vina.

### 2.7 Acquisition and culture of tendon stem cells

Tendon stem cells were obtained from Wuhan Procell (CP-M176). Cells were cultured in an incubator at 37 °C with 5 % CO2. Tendon stem cells were cultured in osteogenic induction medium to induce osteogenic differentiation. The osteogenic induction medium used in this study comprised 10 nM dexamethasone, 50 μg/mL ascorbic acid, 10 mM B-GP disodium, 10 % fetal bovine serum, and high-glucose Dulbecco's Modified Eagle Medium (DMEM, Gibco, MA, USA). IL-1β was used to simulate inflammatory stimulation. The dosage of IL-1β is 10 ng/mL in vitro [16]. And the dosage of Celecoxib is 35umol/L in vitro, In the entire course of the in vitro experiments, IL-1β and Celecoxib were administered only once.

### 2.8 RT-qPCR

Total RNA was extracted and purified from the treated cells using TRIzol Reagent (Invitrogen). Quantification of total RNA was performed based on the A260/A280 absorbance ratio using a DeNovix spectrophotometer. cDNA synthesis was conducted using PrimeScript RT Master Mix (Takara), with the reaction held at 37 °C for 15 min, followed by 5 min at 85 °C. Quantitative real-time PCR (RT-qPCR) was then carried out using SYBR Green qPCR (Applied Biosystems) and the StepOnePlus Real-Time PCR System, in accordance with the established protocols. The primer sequences used in this study are listed below.

**Table:** 

Genes	Sequence 5’ -3′
CCND1-mouse-F	GCGTACCCTGACACCAATCTC
CCND1-mouse-R	ACTTGAAGTAAGATACGGAGGGC
Mouse actin beta-RT-F	GTCCCTCACCCTCCCAAAAG
Mouse actin beta-RT-R	GCTGCCTCAACACCTCAACCC
OCN-mouse-F	GAACAGACAAGTCCCACACAGC
OCN-mouse-R	TCAGCAGAGTGAGCAGAAAGAT
RUNX2-mouse-F	GACTGTGGTTACCGTCATGGC
RUNX2-mouse-R	ACTTGGTTTTTCATAACAGCGGA

### 2.9 Alizarin red staining (ARS)

After a 21-day period dedicated to osteogenic induction, the cells underwent ARS. Initially, each well received 1 mL of PBS, which was swiftly aspirated after a minute. The cells were then subjected to fixation with 1 mL of either 70 % ethanol or 10 % neutral buffered formalin for 30 min at 37 °C or room temperature. Upon removal of the fixative, wells were rinsed with 1 mL of a washing solution. Subsequently, 3 mL of 0.1 % Alizarin Red solution (Cyagen Biosciences) was applied to each well for 15–20 min at 37 °C to stain the cells. Post-staining, the cells were rinsed twice with the washing solution before being examined under a microscope.

### 2.10 Alkaline phosphatase (ALP) staining

After a 14-day osteogenic culture, ALP staining was conducted. Initially, 40 μL of reagent A was combined with 1 mL of reaction buffer, followed by the addition of 40 μL of reagent B to complete the reaction mixture, yielding the working solution ready for immediate use (Beyotime Biotechnology). Cells were then rinsed with 1 mL of PBS per well, removed after a minute, and the wells were subjected to a double wash. A 500 μL aliquot of fixative was added to each well for a 30-min fixation period at 37 °C or ambient temperature. The prepared reaction mixture was dispensed into each well, allowing the cells to develop color for 30 min under the same temperature conditions. Finally, each well received 1 mL of a washing solution, followed by two rounds of washing before microscopic examination.

### 2.11 Transfection

In this experiment, we utilized Lipofectamine 2000 reagent (Invitrogen, Carlsbad, CA, USA) for the transfection of tendon stem cells to investigate the function of CCND1. Initially, we prepared the CCND1-pcDNA3.1 plasmid and siCCND1 small interfering RNA, and mixed them with the transfection reagent according to the manufacturer's instructions to form a transfection complex. Subsequently, this complex was added to tendon stem cells at a final concentration of 100 nM to achieve efficient transfection. Within 48 h post-transfection, the cells were continuously cultured in medium containing 10 % FBS to maintain their growth and transfection efficiency. The transfection efficiency was assessed by fluorescent microscopy observation and molecular biology methods. After 48 h, the cells were collected for further experiments.

### 2.12 Statistical analysis

Statistical analyses were performed using SPSS software, version 25.0. Results are expressed as mean ± standard deviation (x ± s). Comparisons among multiple independent groups were conducted using one-way analysis of variance (ANOVA). Pairwise comparisons between groups were performed using the LSD-t test. A P-value of <0.05 was considered statistically significant.

## 3 Results

### 3.1 Identify potential target molecules through which Celecoxib exerts its therapeutic effects on HO

**Fig. 2:**
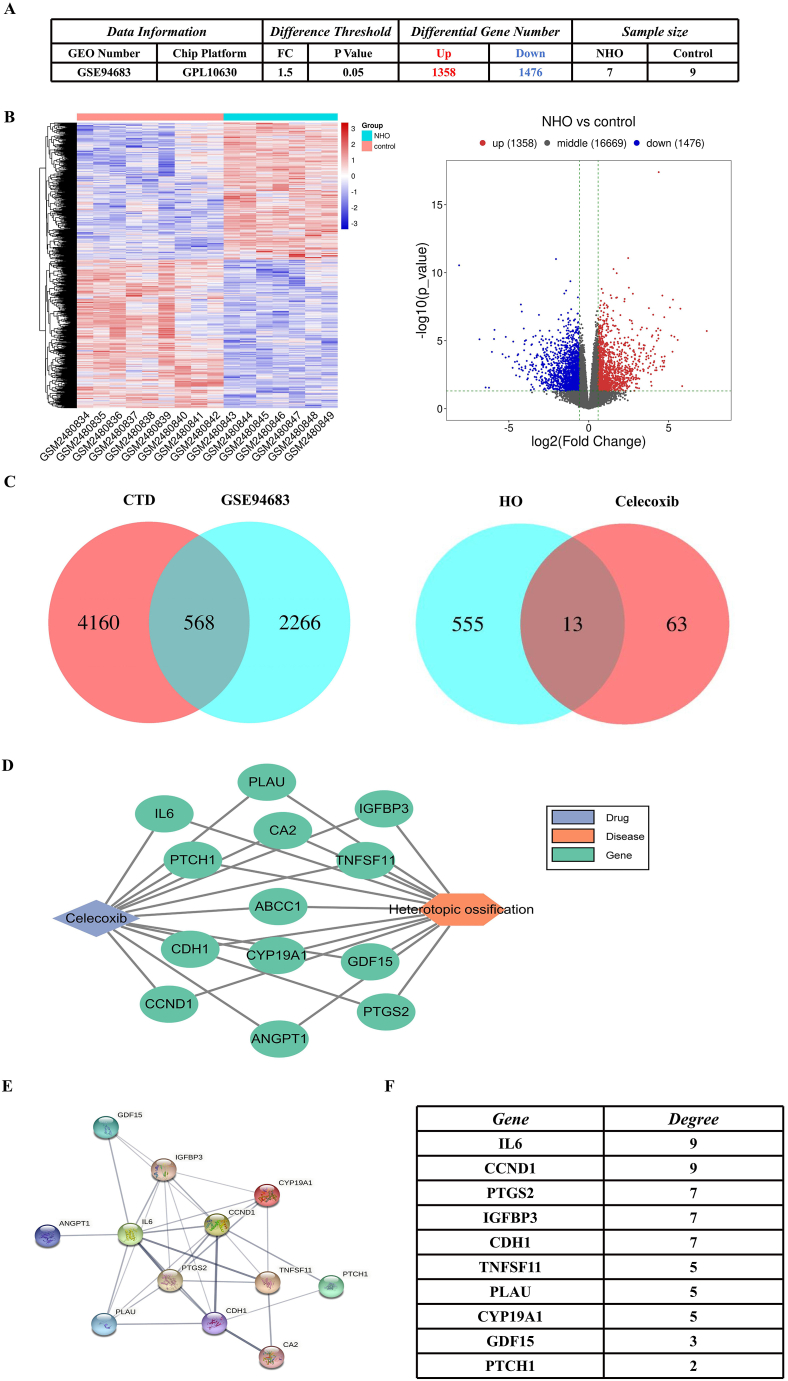
Construct Drug-Target-Disease network and analyze the PPI network **(A)** GEO dataset differential gene expression results summary table. **(B)** Heat map and volcano map of differential genes. **(C)** Intersection Venn diagram of heterotopic ossification target genes in the CTD database and differentially expressed genes in GEO data, obtaining genes related to heterotopic ossification; Intersection Venn diagram of heterotopic ossification-related genes and Celecoxib-related genes. **(D)** Drug-Target-Disease network. E. PPI network. F. Table of gene connectivity in PPI network.

Our analysis of the GSE94683 dataset from the GEO database yielded a significant set of 2834 differentially expressed genes, which were pivotal in understanding the molecular landscape of HO. The heatmap visualization distinctly categorized these genes into two groups: 1358 genes (red dots) that were positively associated with HO development, and 1476 genes (blue dots) that showed a negative correlation (Fig. 2A–B). Further refinement of these findings was achieved by intersecting our differentially expressed genes with the 2266 HO-associated target genes predicted by the CTD database. This strategic approach identified 568 genes as potential disease targets, significantly narrowing down the candidates for further investigation. In pursuit of therapeutic targets, we explored the STITCH database to identify potential Celecoxib targets related to HO. The intersection of these datasets led to the discovery of 13 genes that may underlie the therapeutic effects of Celecoxib on HO (Fig. 2C). These genes were then integrated into Cytoscape software to construct a comprehensive drug-target-gene-disease network, providing a visual representation of the complex interactions at play (Fig. 2D). To delve deeper into the protein-protein interaction (PPI) network, we utilized the STRING database. After a meticulous curation process that involved the exclusion of nodes with low connectivity, we retained 12 targets with a confidence score exceeding 0.4. This stringent criterion allowed us to elucidate the PPI relationships among these targets (Fig. 2E). Moreover, the connectivity of the top ten genes within the PPI network was quantified, highlighting the genes with the highest number of direct interactions (Fig. 2F).

### 3.2 GO and KEGG enrichment analysis of PPI network

**Fig. 3:**
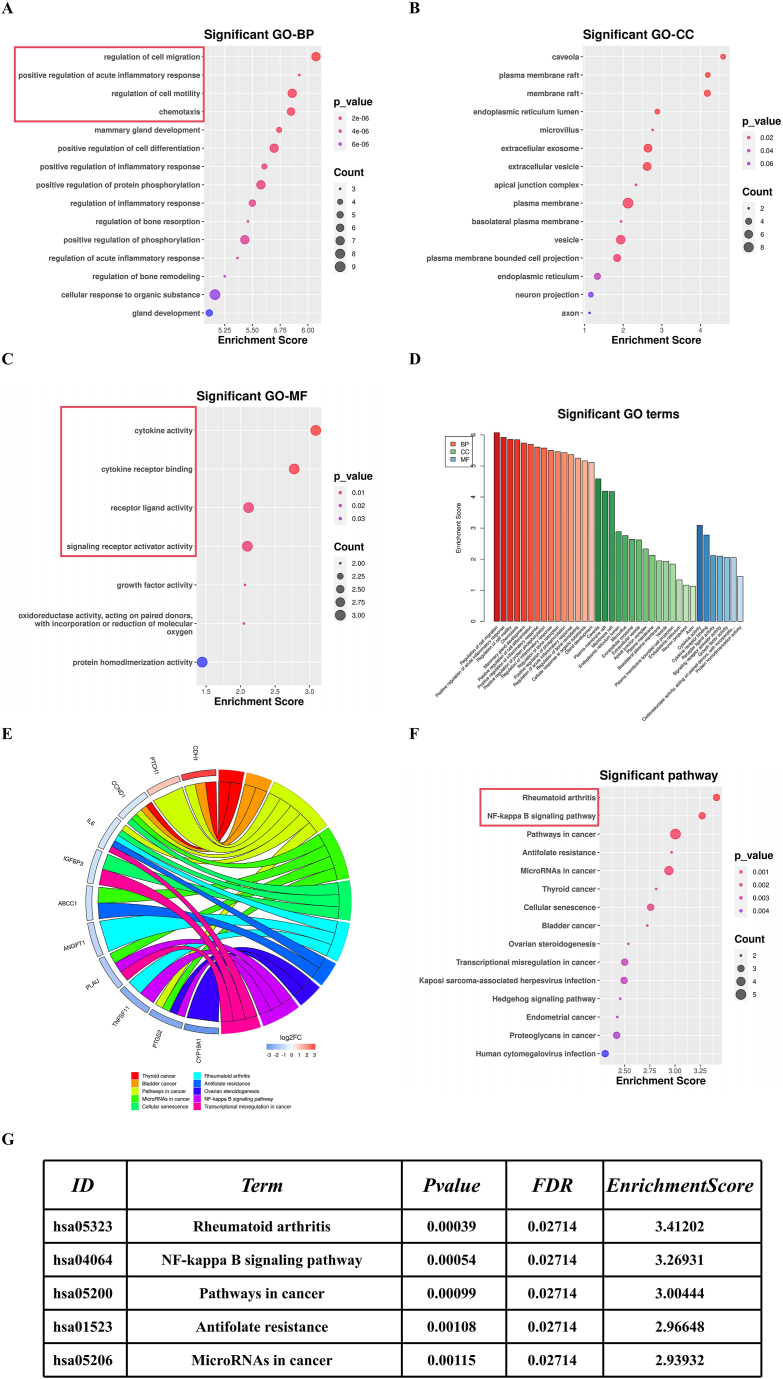
GO and KEGG enrichment analysis of PPI network **(A)** GO enrichment analysis of biological processes. **(B)** GO enrichment analysis of cell components. **(C)** GO enrichment analysis of molecular function. **(D)** GO analysis overview. **(E)** Circular diagram results of KEGG analysis for the top 10 genes with the highest connectivity. **(F)** Bubble chart of KEGG analysis for the Drug-Target-Disease network. **(G)** Data table of the top 5 enriched signaling pathways from KEGG analysis.

Genes in the drug-target-disease network were subjected to GO and KEGG enrichment analysis to further understand the biological processes and signaling pathways primarily involved in Celecoxib's treatment of HO. GO analysis included three categories: Biological Process (BP) (Fig. 3A), Cellular Component (CC) (Fig. 3B), and Molecular Function (MF) (Fig. 3C). BP primarily related to Regulation of cell migration, Positive regulation of acute inflammatory response, Regulation of cell motility, and Chemotaxis. It has been reported that in the early stages of trauma-induced HO, tendon stem cells and mesenchymal stem cells migrate to injured muscles, apart from repairing tendon cells, they often differentiate into osteoblasts under the influence of the local inflammatory microenvironment, ultimately leading to the occurrence and development of HO. MF mainly involves Cytokine activity, Cytokine receptor binding, Receptor ligand activity, and Signaling receptor activator activity. This enrichment result corroborates the BP findings, suggesting that Celecoxib exerts its therapeutic effect by acting on cytokine-activated signaling pathways involved in the progression of HO. The CC enrichment results support the significant activation of functional phenotypes such as the binding of cytokines to their corresponding receptors, as indicated in the MF (Fig. 3D). KEGG results indicate significant enrichment of genes in the drug-target-disease network in pathways like Rheumatoid arthritis and NF-kappa B signaling (Fig. 3E). Rheumatoid arthritis and the NF-kappa B signaling pathway, as classical immune-related and inflammation-related signaling pathways, have been reported in multiple studies to play a significant role in HO (Fig. 3F). Our enrichment results suggest that Celecoxib likely exerts its therapeutic effects on HO by acting on well-researched pathways like Rheumatoid arthritis and NF-kappa B, consistent with our GO analysis findings (Fig. 3G). Combined with relevant literature, these results further validate that the genes in our drug-target-disease network are likely the action targets of Celecoxib in the treatment of HO, underscoring the practical significance of our network pharmacology analysis results.

### 3.3 Hub genes exhibit good binding affinity with Celecoxib

**Fig. 4:**
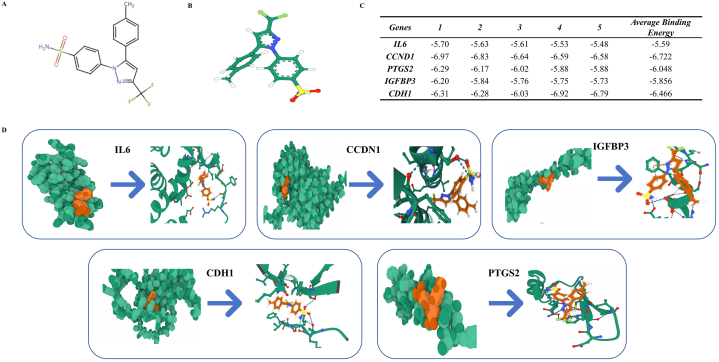
Molecular docking experimental results of hub genes **(A)** Chemical structure of Celecoxib. **(B)** 3D structure of the Celecoxib molecule. **(C)** Table of binding energy values between Hub Genes and the Celecoxib molecule. **(D)** Spherical molecular binding diagrams of each hub gene with Celecoxib and detailed images of the binding sites.

Upon conducting PPI network topology analysis, GO, and KEGG analyses, we identified key hub genes, including IL6, CCND1, PTGS2, IGFBP3, and CDH15, which are likely pivotal in the pathogenesis of HO. These genes were recognized for their central roles within the regulatory network. To substantiate the efficacy of these selected hub genes, we proceeded with molecular docking experiments to explore their interactions with Celecoxib. Initially, the molecular structure of Celecoxib was sourced from Pubchem (Fig. 4A–B). Subsequently, the target protein molecular structures were retrieved from the PDB website. Utilizing Autodock Vina software, we achieved the molecular docking results (Fig. 4D). Our findings revealed that each of the hub genes demonstrated a strong binding affinity to Celecoxib, suggesting that these genes are potential targets through which Celecoxib may exert its therapeutic effects on HO (Fig. 4C). This discovery not only sheds new light on the mechanism of action of Celecoxib but also offers valuable insights for potential therapeutic strategies against HO.

### 3.4 CCND1 is highly expressed in HO, promoting its development in the early stages and inhibiting it in the later stages, and Celecoxib can upregulate the expression level of CCND1

**Fig. 5:**
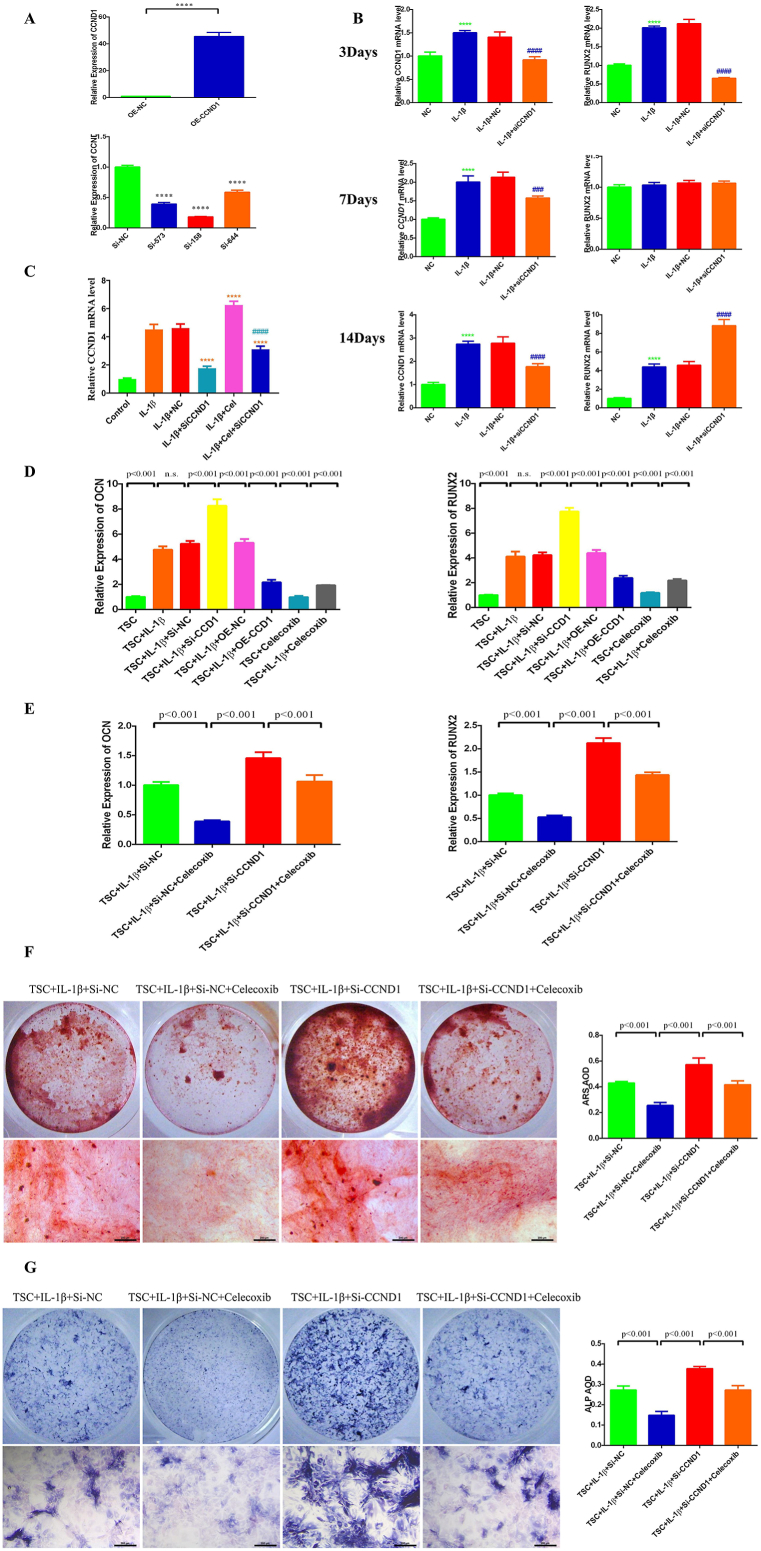
Exploring the mechanism of CCND1 as a Celecoxib target in HO through validation experiments **(A)** Validation of the efficacy of CCND1 overexpression and knockdown plasmids. Data are means ± SD (n = 5) ****p < 0.0001. * compared with Si-NC group. **(B)** Changes in the transcription levels of CCND1 and OCN at 3 days, 7 days, and 14 days time points. Data are means ± SD (n = 5) ***p < 0.001, ****p < 0.0001, ^###^*p* < 0.001, ^####^*p* < 0.0001. * compared with NC group, ^#^ compared with IL-1β+NC group. **(C)** Exploring the regulatory mechanism of Celecoxib on CCND1 through RT-qPCR experiments. ****p < 0.0001, ^####^*p* < 0.0001. * compared with IL-1β+NC, ^#^ compared with IL-1β +Cel group.**(D)** Investigate the relationship between Celecoxib treatment for heterotopic ossification and the expression level of CCND1 using RT-qPCR **(E)** Utilization of RT-qPCR based rescue experiments to validate the dependency of Celecoxib's therapeutic efficacy in HO on the molecular activity of CCND1. Data are means ± SD (n = 5). **(F)** Utilization of ARS based rescue experiments to validate the dependency of Celecoxib's therapeutic efficacy in HO on the molecular activity of CCND1. Data are means ± SD (n = 5). Scale bar = 200 μm. **(G)** Utilization of ALP based rescue experiments to validate the dependency of Celecoxib's therapeutic efficacy in HO on the molecular activity of CCND1. Data are means ± SD (n = 5). Scale bar = 200 μm.

In molecular docking experiments, CCND1 exhibited a more prominent binding ability among the various Hub genes. CCND1 is a cell cycle regulatory protein, playing a vital role in the transition from the G1 to S phase of the cell cycle [17], studies have shown that mutations or overexpression of the CCND1 gene are associated with the occurrence and development of cancer [[18], [19], [20]]. However, whether CCND1 plays a crucial role in HO is currently unclear, with no studies on its mechanism of action, warranting further investigation. Therefore, we aim to verify the role of the Hub gene CCND1 in HO, after constructing CCND1 overexpression and knockdown plasmids (Fig. 5A), we conducted RT-qPCR experiments on tendon stem cells at different culture time points. The results showed that the expression level of CCND1 was significantly increased at all stages, this increase was accompanied by an elevation in the expression level of the osteogenic chemotactic factor RUNX2. Notably, knocking down CCND1 at the 3-day time point could reverse the elevated expression of RUNX2, while knocking down CCND1 at the 14-day time point further promoted the increase in RUNX2 expression (Fig. 5B), this indicates that CCND1 plays different roles at various stages of HO, promoting its onset in the early stages and inhibiting it in the later stages. To observe the regulatory effect of Celecoxib on CCND1, we conducted RT-qPCR experiments on tendon stem cells at the 14-day time point, the results showed that Celecoxib could upregulate the expression level of CCND1, knocking down CCND1 on this basis could reverse the upregulation effect of Celecoxib (Fig. 5C), further observation of the regulation of osteogenic markers by CCND1 and Celecoxib revealed that knocking down CCND1 significantly upregulated the expression levels of osteogenic factors OCN and RUNX2. Celecoxib effectively inhibited the expression levels of OCN and RUNX2, while overexpression of CCND1 mimicked the effect of Celecoxib, significantly inhibiting the expression levels of OCN and RUNX2 (Fig. 5D). These results are consistent with our conclusions.

To investigate whether Celecoxib exerts its therapeutic effect on HO by acting on CCND1, we designed a rescue experiment. The RT-qPCR experiments showed that administering Celecoxib significantly reduced the expression levels of OCN and RUNX2. Knocking down CCND1 on this basis could reverse the effect of Celecoxib alone, promoting osteogenic differentiation in tendon stem cells (Fig. 5E). The ARS (Fig. 5F) and ALP (Fig. 5G) staining results were consistent with the RT-qPCR findings, demonstrating that Celecoxib alone could inhibit osteogenic differentiation in tendon stem cells. Meanwhile, knocking down CCND1 could reverse this inhibitory effect. These results suggest that Celecoxib's inhibitory effect on HO is dependent on CCND1.

## 4 Discussion

HO refers to the atypical bone formation occurring in muscles or connective tissues, resulting in the formation of mature lamellar bone, and is a pathological bone formation phenomenon [21]. HO, as a complication of central nervous system diseases, was first reported by Dejerine and Ceillier in 1918. Subsequently, in 1968, Roberts conducted studies on HO occurring around the joints of patients after brain trauma [22]. This gained the attention of clinicians and was gradually studied as a complication in clinical practice, and related fundamental mechanism research also developed. The pathogenesis of HO is complex, involving genetics and gene mutations, participation of osteogenic precursor cells, upregulation of BMP signaling pathways, and overexpression of BMP [23]. Current treatments for HO include pharmacotherapy, radiotherapy, physiotherapy, and surgical intervention [[24], [25], [26], [27]]. Since the exact mechanism of HO formation is still unclear, there is no definitive medication for its treatment [28]. The reason is that the currently isolated and purified drug targets are diverse, making it impossible to select a definitive anti-HO medication through clinical practice. Celecoxib is a highly selective NSAID, whose widely recognized mechanism of action mainly involves targeted inhibition of cyclooxygenase-2, suppressing the production of inflammatory prostaglandins in the early stages of trauma, reducing local inflammation, and preventing the conversion of mesenchymal stem cells into osteoblasts to prevent traumatic HO [5,6]. The efficacy of Celecoxib is comparable to that of the commonly used Indomethacin, but it has much fewer gastrointestinal side effects [7,8].

Network pharmacology is an emerging discipline that combines pharmacology, computer science, and information networks on the basis of systems biology [29]. It emphasizes exploring the relationships between drugs, targets, and diseases from the perspective of biological networks [30]. Compared to traditional pharmacology, network pharmacology can comprehensively explain the potential mechanisms of drug action using scientific analytical methods. It provides new support for rational clinical medication, elucidating the overall mechanisms of action, and analyzing drug combination patterns [31]. In this study, we used network pharmacology methods to analyze the potential therapeutic targets of Celecoxib in treating HO. We first obtained 568 HO-related target genes through the CTD and GEO databases. Then, we acquired all 76 target genes of Celecoxib from the STITCH database. After intersecting them, we identified 13 potential therapeutic target genes of Celecoxib for HO. Subsequently, to clarify the multiple mechanisms of Celecoxib in treating HO, we conducted GO and KEGG enrichment analysis on these targets. The GO results indicated that the potential targets of Celecoxib in treating HO are closely related to the following molecular functions: cytokine activity, cytokine receptor binding, receptor ligand activity, signaling receptor activator activity; and closely related to the following biological processes: regulation of cell migration, positive regulation of acute inflammatory response, regulation of cell motility, chemotaxis. The CC enrichment results supported the conclusion of significant activation of functional phenotypes such as the binding of cytokines to their corresponding receptors at the cellular structural level. The KEGG enrichment analysis results showed that the signaling pathways involving these targets in HO mainly include Rheumatoid arthritis and NF-kappa B signaling pathway. Rheumatoid arthritis and the NF-kappa B signaling pathway, as classic immune-related and inflammation-related signaling pathways, have been reported to play significant roles in HO in multiple studies. Huang et al. have demonstrated the important role of the NF-kappa B signaling pathway in HO, and palovarotene can alleviate HO by blocking the NF-kappa B signaling pathway. Palovarotene can weaken tendon stem cell-induced HO by downregulating the synergistic effect of NF-kappa B and Smad signaling pathways following inflammatory microenvironment stimulation [16,32,33]. Additionally, Ju et al. found that the NF-kappa B/p53 signaling pathway plays an important role in the occurrence and development of HO in models such as traumatic brain injury, burns, and tendon transection, Pyrrolidine dithiocarbamate (PDTC) inhibition of the NF-kappa B signaling pathway can significantly reduce the expression level of p53 and the size of HO [34].

Next, we constructed a PPI network using the target set and identified Hub genes including IL6, CCND1, PTGS2, IGFBP3, CDH15, based on centrality through topological analysis. IL-6 is a pleiotropic cytokine that plays an important role in inflammatory responses [35]. IL-6 is known to promote the maturation and activation of osteoclasts [36,37] and affect osteoblasts [38]. CCND1 is a cell cycle protein that regulates cells through the transition from G1 to S phase, cell proliferation, and differentiation [17]. Prostaglandin-Endoperoxide Synthase 2 (PTGS2) is a key enzyme in prostaglandin biosynthesis associated with physiological stress, such as infection and inflammation [39,40]. Insulin-like Growth Factor Binding Protein 3 (IGFBP3) is a member of the IGFBP family, a p53 tumor suppressor regulatory protein, and also acts as an important IGF carrier in circulation, regulating its activity [41]. CDH15 is a member of the cadherin gene superfamily, encoding calcium-dependent intercellular adhesion glycoproteins [42]. To validate the effectiveness of the Hub genes, we conducted molecular docking experiments, where all the Hub genes demonstrated good binding affinity with Celecoxib, especially CCND1. Therefore, we conducted a series of validation experiments focusing on CCND1. We found that CCND1 is highly expressed during the course of HO and plays different roles at different stages, promoting HO in the early stages and inhibiting it in the later stages, and Celecoxib can effectively upregulate the expression level of CCND1. After the early stages of HO, overexpression of CCND1 and administration of Celecoxib significantly inhibited the expression of osteogenic markers, suppressing the osteogenic tendency of tendon stem cells. We also found that Celecoxib's inhibitory effect on HO depends on CCND1, indicating the significance of our network pharmacology analysis.

Our study not only uncovers the pharmacological mechanisms of Celecoxib beyond being a cyclooxygenase-2 inhibitor but also delves into the pivotal role of CCND1 in the progression of HO. By elucidating the dual role of CCND1 in promoting HO in the early stages and inhibiting it in the later stages, we believe this offers a new perspective for the development of stage-specific therapeutic strategies. Although our study provides new insights into the clinical treatment of HO, we acknowledge the limitations, particularly the lack of experimental data to validate the mechanisms in both animal and human subjects. To overcome these limitations, we plan to further validate the efficacy of CCND1 as a therapeutic target through animal models in future research and explore its potential applications in humans.

Future therapeutic directions may include the development of drugs capable of modulating the activity of CCND1 to achieve precise intervention at different stages of HO. In particular, the potential application of Celecoxib, which can upregulate the expression of CCND1, in the treatment of HO warrants further exploration. We propose evaluating the combined therapeutic effects of Celecoxib with other drugs in clinical trials to achieve better therapeutic outcomes. Additionally, monitoring the expression levels of CCND1 in patients will assist physicians in formulating personalized treatment plans, optimizing the timing and duration of treatment.

## Funding

This work is supported by 10.13039/501100008750Shanghai Hospital Development Center Foundation (SHDC22024242), Shanghai Pujiang Programme (23PJD079), 10.13039/501100003399Shanghai Municipal Science and Technology Commission Natural Science Fund (20ZR1443200), 10.13039/100017950Shanghai Municipal Health Commission Clinical Research General Project Fund (202240132), Program for Research-oriented Physician of Shanghai Tenth People's Hospital (2023YJXYSA004).

## Data availability statement

Data will be made available on request from the corresponding author.

## CRediT authorship contribution statement

**Wei Liu:** Writing – original draft, Project administration, Formal analysis, Data curation, Conceptualization. **Junchao Huang:** Writing – original draft, Project administration, Formal analysis, Data curation, Conceptualization. **Jianhai Hu:** Project administration, Formal analysis, Data curation, Conceptualization. **Ziheng Bu:** Project administration, Formal analysis, Data curation. **Zheng Zhou:** Project administration, Formal analysis, Data curation. **Jianing Yu:** Project administration, Formal analysis, Data curation. **Huajun Wang:** Supervision. **Xinbo Wu:** Supervision, Funding acquisition. **Peng Wu:** Supervision, Funding acquisition.

## Declaration of generative AI and AI-assisted technologies in the writing process

During the preparation of this work the authors used ChatGPT4 in order to refine and enhance the language and clarity of the manuscript. After using this tool, the authors reviewed and edited the content as needed and takes full responsibility for the content of the publication.

## Declaration of competing interest

The authors declare that they have no known competing financial interests or personal relationships that could have appeared to influence the work reported in this paper.
